# Adipose derived mesenchymal stem cells transplantation via portal vein improves microcirculation and ameliorates liver fibrosis induced by CCl4 in rats

**DOI:** 10.1186/1479-5876-10-133

**Published:** 2012-06-26

**Authors:** Yu Wang, Fan Lian, Jiaping Li, Wenzhe Fan, Hanshi Xu, Xiuyan Yang, Liuqin Liang, Wei Chen, Jianyong Yang

**Affiliations:** 1 Department of Interventional Oncology, the First Affiliated Hospital of Sun Yat-sen University, No. 58, Zhongshan 2nd Road, Guangzhou, 510080, China; 2 Department of Rheumatology & Clinical Immunology, the First Affiliated Hospital of Sun Yat-sen University, No. 58, Zhongshan 2nd Road, Guangzhou, 510080, China; 3 Department of Interventional Radiology, the First Affiliated Hospital of Sun Yat-sen University, No. 58, Zhongshan 2nd Road, Guangzhou, 510080, China; 4 Department of Medical Imaging, the First Affiliated Hospital of Sun Yat-sen University, No. 58, Zhongshan 2nd Road, Guangzhou, 510080, China

**Keywords:** Adipose derived mesenchymal stem cell, Liver fibrosis, Portal venous transplantation, Computed tomography perfusion scan, Vascular endothelial growth Factor

## Abstract

**Introduction:**

Adipose derived mesenchymal stem cells (ADMSCs), carrying the similar characteristics to bone marrow mesenchymal stem cells, only much more abundant and easier to obtain, may be a promising treatment for liver fibrosis. We aim to investigate the therapeutic potential of ADMSCs transplantation in liver fibrosis caused by carbon tetrachloride (CCl_4_) in rats as well as its underlying mechanism, and to further explore the appropriate infusion pathway.

**Methods:**

ADMSCs were isolated, cultured and identified. Placebo and ADMSCs were transplanted via portal vein and tail vein respectively into carbon tetrachloride (CCl_4_)-induced liver fibrosis rats. Computed tomography (CT) perfusion scan and microvessel counts were performed to measure the alteration of liver microcirculation after therapy. Liver function tests and histological findings were estimated.

**Results:**

CT perfusion scan shown significant decrease of hepatic arterial perfusion index, significant increased portal vein perfusion, total liver perfusion in rats receiving ADMSCs from portal vein, and Factor VIII (FVIII) immunohistochemical staining shown significant decrease of microvessels in rats receiving ADMSCs from portal vein, indicating microcirculation improvement in portal vein group. Vascular endothelial growth Factor (VEGF) was significantly up-regulated in fibrosis models, and decreased after ADMSCs intraportal transplantation. A significant improvement of liver functional test and histological findings in portal vein group were observed. No significance was found in rats receiving ADMSCs from tail vein.

**Conclusions:**

ADMSCs have a therapeutic effect against CCl_4_-mediated liver fibrosis. ADMSCs may benefit the fibrotic liver through alteration of microcirculation, evidenced by CT perfusion scan and down-regulation of VEGF. Intraportal transplantation is a better pathway than tail vein transplantation.

## Introduction

Liver fibrosis representing the final common pathway of all chronic hepatic injury is a worldwide health problem [1,2]. Limited therapy would repair the malformation or reverse the fibrosis in progression [3], and currently the only effective available treatment for end stage hepatic fibrosis is transplantation [4].

However, a series of problems such as organ shortage, recrudescence of the original disease in transplant recipients and transplantation complications underlines the need for other antifibrotic therapeutic approaches.

Bone marrow-derived mesenchymal stem cells (MSCs) have been reported to benefit in the prevention of pulmonary fibrotic lesions [5], or ameliorate changes of liver function tests in experimental fibrosis models [6].

However, the estimated frequency of BM-MSCs in bone marrow is relatively low [7], and the interest of using other sources of MSCs has been growing.

Adipose derived mesenchymal stem cells (ADMSCs), as a source of adult mesenchymal stem cells, display similar multiple-linage differentiating potentials to bone marrow MSCs [8-11]. ADMSCs are much more abundant than BM-MSCs and easy to obtain from liposuction aspirates or excised fat. The yield from one gram adipose tissue is approximately 5,000 to 20,000 stem cells, whereas the yield from 1 ml BM-MSCs is only 2% to 20% of ADMSCs, which makes ADMSCs a promising alternative for cytotherapy.

In the present study, we sought to test the hypothesis that transplantation of ADMSCs could effectively counteract liver fibrogenesis induced by CCl4 in Sprague–Dawley (SD) rats, improve microcirculation as well as liver function. We further investigated whether portal vein infusion was an ideal route for ADMSCs implantation.

## Materials and methods

### Isolation, culture and identification of adipose-derived stem cells from rat

The Sprague–Dawley (SD) rats (weight around 200 g) housed under standard conditions were anesthetized and underwent lipo-dissection from inguinal fat pad. ADMSCs were isolated based on a published method described by Safford et al [12]. All experiments were approved by the Animal Experiment Committee of our University, following the Guide for the Care and Use of Laboratory Animals (National Academic Press, USA, 1996).

100 mg adipose tissue was taken from SD rats and digested in Hank's balanced salt solution containing 0.1% collagenase type I (Gibco, Carlsbad, CA, USA) for 1 hour at 37 °C in a centrifuge tube after carefully dissociation, followed by filtering with a 100 μm cell strainer and centrifugation at 1700 rpm for 10 minutes at room temperature, the supernatant was discarded. Dulbecco’s modified Eagle’s medium (DMEM; Gibco) supplemented with 10% fetal bovine serum (FBS; Gibco) were added. The resuspended cells were transferred to a culture flask and incubated in 5% CO_2_ at 37 °C. The medium was first replaced at 48 hours, and the non-adherent cells were removed. Rat ADMSCs at the 3^rd^-5^th^ passage were identified and cultured for future experiments.

The adherent cells at 3 ^rd^ -5^th^ were trypsinized and centrifuged, fixed in neutralized 2% paraformaldehyde solution for 30 minutes. The fixed cells were washed twice and resuspended in PBS, incubated with FITC-labeled anti-rat CD34, CD45, CD29, CD44 and CD90 [13,14] (all from Santa Cruz Biotech., CA) for 30 minutes. Positive cells were counted by flow cytometry with FACscan flow cytometer (Becton Dickinson, San Jose, CA).

ADMSCs at 5^th^ were resuspended at 2 × 10^6^ cells/ml in saline for autologous transplantation.

### Animals and treatments

Pathogen-free male Sprague–Dawley (SD) rats (weight around 200 g) purchased from the Sun Yat-sen University Animals Technology (Guangzhou China) were housed in controlled temperature of 25 °C, relative humidity ~70%, and a 12 h light/dark cycle. All experiments were approved by the Animal Experiment Committee of our University, following the Guide for the Care and Use of Laboratory Animals (National Academic Press, USA, 1996). Hepatic fibrosis was induced by intraperitoneal injections of CCl_4_ twice a week for 12 weeks. The initial dose of CCl_4_ (diluted 1:1 in olive oil) was 5 mL/kg, and each subsequent dose was 3 mL/kg (diluted 1:1 in olive oil) [15]. After 12 weeks of CCl_4_ injury, all rats were submitted to liver function blood tests. Samples of 10 normal rats were used to define the normal control range of alanine aminotransferase (ALT) and aspartate aminotransferase (AST), albumin.

The rats presenting abnormal ALT, AST and albumin were randomly assigned to three groups forty-eight hours after the last CCl_4_ injection. Each group included 2 randomly picked rats for fibrosis model confirmation and 15 rats for further experiments: A) the control group without ADMSCs: 2 × 10^6^ irrelevant cells (BRL-3A, a rat liver cell line) was injected in portal vein. B) The portal vein group: rats were anesthetized and exposed with an upper midline incision inferiorly from the xiphoid, and the abdominal cavity was exposed with a retractor. Portal vein was gently isolated and directly injected with 2 × 10^6^ ADMSCs using a 25-gauge needle connected to a 2 ml syringe [15,16]. The abdominal wall was closed after bleeding stopped by pressure. C) The tail vein group: 2 × 10^6^ ADMSCs were injected in the tail vein.

CCl_4_ administration was continued after ADMSCs transplantation in case of spontaneous fibrosis recovery [17].

### Computed tomography perfusion imaging

CT perfusion was performed with a 64-detector row CT scanner. (Toshiba Auqillion 64 TSX-101A, Toshiba, Tochigi, Japan). Each rat was stabled with 10% chloral hydrate and inserted with a silicone rubber tube into the tail vein for contrast injection before scanning. Plain CT scan of the abdomen was for localization of the liver, abdominal aorta and portal vein area. Scanning parameters were as follows: 120 kV,80 mA,reconstruction field of view 90 mm, standard reconstruction algorithm, 35 cm display field of view, matrix 512 × 512. A bolus of 0.1 ml/100 g of nonionic iodinated contrast medium containing 300 mg I/ml (Ultravist 300, Bayer Schering, Germany) was injected into the tail vein at a rate of 1.0 ml/s and continued for 80 seconds with. 0.5 second per rotation, with four 2-mm sections acquired per rotation.

The data were then transferred to an image processing workstation (Vitrea 2, version 4.1.2.0, Vital Images, Minnetonka, MN, USA) and analyzed by two independent radiologists using Ascend CT Perfusion (Dynamic CT Perfusion System, Ascend Medical Technology, Beijing, China)., in which hepatic artery perfusion (HAP), portal vein perfusion (PVP), total liver perfusion (TLP) and hepatic arterial perfusion index (HPI = HAP / TLP, %) were measured [18,19].

The examination was repeated at baseline, 2 days after liver fibrosis model constructed, and 6 weeks after placebo/ADMSCs infusion.

### Reverse transcription PCR (RT-PCR), quantitative PCR (qPCR) and western blot

Total RNA was isolated from snap-frozen rat tissues, first-strand DNA was synthesized as previously described [20], and qPCR was performed (in triplicate reactions) using the SYBR®-Green PCR Master Mix (Applied Biosystems, Applera Deutschland GmbH, Darmstadt, Germany) in the thermocycler ABI PRISM TM7700. For analysis of the VEGF, RT-qPCR was used to detect VEGF mRNA level. Primer sequences for rat VEGF were designed according to Liu et al [21].

Western blotting was used to detect VEGF protein level. Antibodies were purchased from Santa Cruz Biotechnology Inc. (Santa Cruz, CA).

### Histology

Liver samples were obtained 12 weeks after the administration of CCl_4_ to confirm the presence of fibrosis, 18 weeks after CCl_4_ administration for control, and 6 weeks after ADMSCs/placebo injection. Histological analysis was conducted by a blinded fashion pathologist. The left lobe of the liver was formalin-fixed, paraffin-embedded and stained with Hematoxilin & Eosin (HE). Sirius red staining was used to assess extracellular matrix accumulation. Briefly, liver sections cut at 10 μm were fixed with 4% paraformaldehyde. Extent of fibrosis were measured using an Axioplan 2 microscope (Carl Zeiss, Thornwood, NY). Five nonoverlapping fields randomly picked in per section were analyzed at 100× final magnification. Quantification of fibrotic area was expressed as the percentage of stained area in comparison to the total area of fields examined, using Image-Pro Plus 5.0 (Media Cybernetics, Bethesda, MD) image analysis software.

### Immunohistochemistry

Sections from paraffin-embedded tissue (4 μm) were stained with Factor VIII antibody (1:100, Santa Cruz Biotechnology Inc.) at 4 °C overnight, then incubated with biotinylated IgG at room temperature for 60 min. Sections were then incubated with DAB and photographed with an Axioplan 2 microscope (Carl Zeiss, Thornwood, NY). The numbers of vessels less than 100 μm in diameter highlighted by FVIII stained endothelial cells were counted in a blinded fashion. Five sections from each animal were scanned and averaged. The microvessel count per square millimeter after treatment was expressed as fold normal values relative to normal control.

### Hydroxyproline determination

Hepatic hydroxyproline content was quantitated using the chloramine T method as described before [22] with minor modifications. In brief, liver specimens (1 mg) were hydrolyzed in 2 ml of 6 N HCl at 100 °C for 12 h. After centrifugation at 3000 rpm for 10 min, the hydrolysate was neutralized with 6 N NaOH, and centrifuged at 13,000 g for 12 min. Fresh prepared Chloramine T solution (1 ml) were added to the neutralized supernatant (1 ml) and incubated at room temperature for 10 min. Ehrlich’s solution (4 ml) was then added and incubated for 15 min at 65 °C. The absorbance was transferred to a plastic tube after cooling and measured at 560 nm. Hydroxyproline concentration was expressed as micrograms of hydroxyproline per gram of liver.

### Biochemical analysis

Serum was separated by centrifugation within 1 h of blood collection when the rats were sacrificed and stored at -20^0^ C until analyzed. Serum levels of aspartate transaminase (AST), alanine transaminase (ALT), and total bilirubin (T-BiL) were determined using an Automated Chemical Analyzer (7600; Hitachi, Tokyo, Japan) according to the manufacturer’s instructions.

### Statistical analysis

All measurements were repeated three times, and results are expressed as the mean ± SD. Differences between two groups were compared with Student’s t-test. Groups were compared with one-way ANOVA test. P < 0.05 was considered statistically significant. Statistical analysis was run with SPSS 13.0.

## Results

### Isolation and identification of ADMSCs

**Figure 1:**
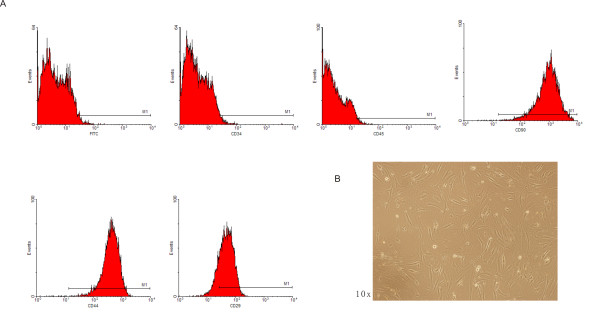
**Isolation and identification of ADMSCs.A**, flow-cytometric analysis of rat ADMSCs. ADMSCs were incubated with FITC-conjugated antibodies. Primary rat ADMSCs positively expressed MSC specific markers CD29 (+), CD44 (+), CD90 (+), but negatively expressed hematopoietic stem cell markers including CD34 (−) and CD45 (−). **B**, rat ADMSCs are spindle-shaped after ten days in culture.

ADMSCs were isolated from SD rat inguinal fat pad, and proliferating cells were enriched as described before. ADMSCs had spindle-shaped morphological feature in ten days culture (Figure 1A).

We measured the expression of surface marker. The fifth passage of ADMSCs expressed CD29, CD44 and CD90, and were negative of CD34 and CD45 (Figure 1B)

### ADMSC transplantation improved microcirculation in fibrotic liver

All rats survived the 12 week CCl_4_ administration. We performed CT perfusion scan on experimental subjects to measure the alteration of liver microcirculation. The measurements of HAP, PVP, TLP and HPI suggested significantly decreased portal venous and total liver perfusion, and significant increase of hepatic arterial to total hepatic perfusion ratio, compared with that of healthy controls, indicating microcirculation impairment in fibrotic models and relative compensation of blood supply with hepatic arterial perfusion (Table 1).

**Table 1:** **Comparison between normal rats and fibrotic models (12 weeks CCl**_**4**_**) on perfusion parameters**

	**HAP**	**PVP**	**TLP**	**HPI**
Normal subjects (n = 12)	26.4 ± 8.2	120.3 ± 39.8	146.7 ± 43.3	18.0 ± 3.7
Fibrotic models (n = 12)	49.2 ± 19.2*	31.4 ± 9.3*	80.5 ± 26.8*	61.1 ± 22.2

Values are expressed as mean ± SD.

*P < 0.05, Fibrotic models versus Normal rats.

PVP: Portal vein perfusion (mL/min per 100 mL); HAP: Hepatic artery perfusion (mL/min per 100 mL).

TLP: Total liver perfusion (mL/min per 100 ml); HPI: Hepatic artery perfusion index (%).

ADMSC transplantation improved microcirculation in fibrotic liver. SD rats with liver fibrosis were randomly divided into three subgroups: one was given irrelevant cells as control; the other two were given the same dosage of ADMSCs, in portal vein and tail vein respectively. After CCl_4_ administration for 18 weeks and ADMSCs/placebo treatment, 6 rats died in the irrelevant cells group (60% survival), 4 rats died in the tail vein group (73% survival), and only 1 rat died in the portal vein group (93% survival).

We performed CT perfusion scan after treatment protocols. HPI were significantly decreased in rats receiving ADMSCs from portal vein (Group B), while PVP and TLP were significantly increased (p < 0.05) compared with baseline. No significance (p > 0.05) was found in rats receiving ADMSCs from tail vein (Group C), though the data seemed change with the same tendency as group B. The data indicated liver microcirculation improvement was significantly observed in ADMSC portal vein group, but neither in ADMSC tail vein group or irrelevant cells portal vein group (Table 2).

**Table 2:** CT perfusion parameters of before and after therapy in test and control group

	**PVP**		**HAP**		**TLP**		**HPI**	
	**Before therapy**	**After therapy**	**Before therapy**	**After therapy**	**Before therapy**	**After therapy**	**Before therapy**	**After therapy**
Irrelevant cells (n = 9)	33.2 ± 8.5	26.5 ± 7.9	53.3 ± 17.0	51.8 ± 17.3	86.5 ± 29.3	78.3 ± 34.5	61.6 ± 19.9	66.1 ± 21.3
Portal vein (n = 14)	33.4 ± 11.0	65.8 ± 15.9*	48.4 ± 19.4	51.2 ± 23.9	81.9 ± 24.4	117.0 ± 37.4*	59.2 ± 20.4	43.7 ± 15.9*
Tail vein	29.2 ± 8.0	33.5 ± 10.0	47.3 ± 22.8	50.4 ± 22.8	76.5 ± 29.6	83.9 ± 32.3	61.8 ± 26.3	60.0 ± 25.5

Values are expressed as mean ± SD.

*P < 0.05, after therapy versus before therapy.

PVP: Portal vein perfusion (mL/min per 100 mL); HAP: Hepatic artery perfusion (mL/min per 100 mL).

TLP: Total liver perfusion (mL/min per 100 ml); HPI: Hepatic artery perfusion index (%).

**Figure 2:**
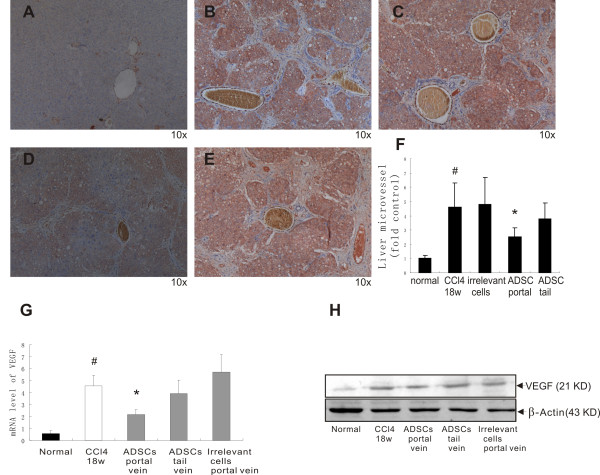
**Agiogenesis after ADMSC transplantation.** Immunohistochemical staining of factor VIII (FVIII) and microvessel counts. **A**, normal control. **B**, 18 weeks of CCl_4_ administration. **C**, portal venous injection of irrelevant cells. **D**, portal vein ADMSC transplantation. **E**, tail vein ADMSC transplantation. **F**, microvessel counts. ADMSCs portal vein transplantation decreases VEGF expression in the fibrotic liver at gene and protein level. **G**, qPCR examination. **H**, Western blotting. *p < 0.05 versus 18 weeks of CCl_4_ administration; #p < 0.05 between normal control and CCl_4_ treatment (18w).

To further explore angiogenesis in fibrotic models, microvessels were highlighted with anti-factor VIII antibody (Figure 2A-E), a specific marker for endothelial cells, and quantified as microvessels counts expressed in fold control (Figure 2F). There was a significant increase of FVIII positive hepatic microvascular staining in CCl_4_ induced cirrhosis models. Hepatic microvessel counts showed substantial decrease of FVIII staining in portal venous ADMSC group. No significant change was observed in tail vein ADMSC group. We examined VEGF expression at gene (Figure 2G) and protein (Figure 2H) level. As an angiogenesis marker, hepatic VEGF up-regulation was detected in CCl_4_ induced fibrotic models. After ADMSCs/placebo treatment, VEGF levels were significantly lower in ADMSC portal vein group than in ADMSC tail vein group or irrelevant cells control portal vein group, suggesting the up-regulation of VEGF triggered by restricted blood supply of liver fibrosis could be partially converted by effective ADMSC transplantation.

### Effects of ADMSCs transplantation on liver function tests

**Figure 3:**
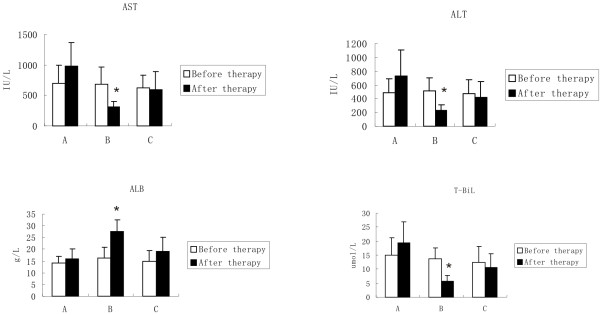
**Liver function test values.** The histograms show AST, ALT, ALB and T-BiL changes before and after placebo/ADMSCs infusion in 3 different groups (**A**, the control group without ADMSCs; **B**, The portal vein group with ADMSCs; and **C**, The tail vein group with ADMSCs). Values are expressed as mean ± SD. *P < 0.05, after therapy versus before therapy.

Pre- and post-therapy laboratory findings suggested the protection capability of ADMSCs. Serum ALT, AST and T-BiL were significantly higher, and ALB was significantly lower in fibrotic models (data not shown). After ADMSCs transplantation (Figure 3), a significant decline in ALT (518 ± 189 U/L versus 229 ± 76 U/L for before and after therapy, *P* < 0.05), AST (689 ± 275 U/L versus 316 ± 87 U/L for before and after therapy, *P* < 0.05) and serum bilirubin (13.6 ± 4.1 umol/L versus 5.6 ± 2.2 umol/L for before and after therapy, *P* < 0.05) and a significant increase of ALB (16.2 ± 4.8 g/L versus 27.4 ± 5.3 g/L for before and after therapy, *P* < 0.05) was observed in portal vein group (Group B). No significant changes were found in saline portal vein group or ADMSC tail vein group.

### Liver fibrosis suppression by ADMSCs transplantation

**Figure 4:**
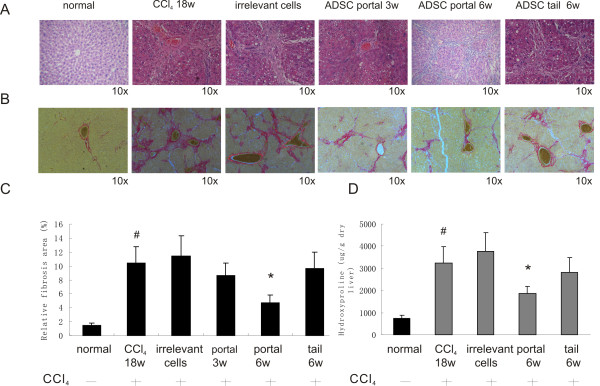
**Portal venous transplantation of ADMSC inhibits CCl**_**4**_**induced hepatic fibrosis**. Collagen fibers deposition was evaluated by **A**, H&E staining and **B**, Sirius red staining. **C**, Sirius red staining quantification. **D**, Hepatic hydroxyproline content was measured. Portal venous transplantation of ADMSC reversed fibrosis induced by CCl_4_ injection, whereas tail vein transplantation of ADMSC failed to reduce the fibrosis significantly. *p < 0.05 versus 18 weeks of CCl_4_ administration; #p < 0.05 between normal control and CCl_4_ treatment (18w).

Liver fibrosis induced by CCl_4_ and after ADMSCs transplantation was evluated using H&E staining (Figure 4A), Sirius red staining (Figure 4B), Sirius red staining quantification (Figure 4C) and hydroxyproline measurement (Figure 4D). In normal control, collagen fibers were observed only in the periportal area. CCl_4_ treatment aroused fibrotic septa, pseudolobuli, hepatic steatosis and necrosis of liver cells. Sirius red stained collagen was significantly increased after CCl_4_ treatment. Portal venous implantation of ADMSCs significantly reduced steatosis lesions, alleviated necrosis, and relatively suppressed bridging fibrotic septa. Three weeks after implantation, the Sirius red positive area were gradually reduced. Six weeks after implantation, a significant decrease in collagen was noted in the ADMSCs treated group compared to the untreated rats (4.73% ± 1.17% vs 11.43% ± 3.16%, P < 0.05). Tail vein implantation of ADMSCs reduced steatosis lesions, but failed to decrease the collagen content significantly as expressed by quantification of Sirius red staining. Hydroxyproline content changes paralleled the extent of fibrosis. ADMSCs portal venous transplantation blocked the CCl_4_ induced hydroxyproline content increase in liver, indicating significant decrease of collagen deposition. The amount of collagen slightly dropped in tail vein transplantation group, but no significance was observed.

## Discussion

MSCs are currently investigated as an alternative therapy for liver transplantation in end-stage liver diseases including severe liver fibrosis. MSCs have also been proved to be a potential solution in severe liver injury such as ischemia-reperfusion injury [23], acute liver failure [24], or hepatocellular carcinoma [25].

Studies have shown that MSCs existed in various human tissues [26,27], and have the ability to differentiate into hepatocyte in inducing medium [28,29].

Carbon tetrachloride (CCl_4_)-induced liver fibrosis in rodents was reported to be prevented receiving MSC transplantation [30-32].

Adipose tissue derived mesenchymal stem cells (ADMSCs), as a source of MSC, exhibit similar differentiation and multi-functional abilities as bone marrow MSC, are better immuno-compatible, much easier to isolate and expand. ADMSCs may provide a new approach for hepatic fibrosis therapy.

In the present study, ADMSCs were isolated from SD rat inguinal fat pad and identified by surface antigen tests. Purified ADMSCs positively expressed MSC markers CD29, CD44 and CD90, whereas negatively expressed hematopoietic stem cell markers CD34 and CD45. Previous studies have proved the long term survival of ADMSCs engraftment in liver parenchyma [8,33].

In the present study, when transplanted with ADMSCs, CCl_4_-induced fibrosis SD rats exhibited microcirculation changes under CT perfusion scan. Significant increase of blood supply was observed in portal vein group (Group B). Interestingly, no significance was found in tail vein group (Group C). Liver function tests and histological examination revealed the same tendency, which was not well in accordance with the study by Rabani V et al, who reported tail vein transplantation of MSCs helped to reduce liver fibrosis [34].

CT perfusion was a non-invasive technique to sensitively detect hemodynamic changes in liver tissue. Since Miles et al. reported the possibility to analyze hepatic perfusion using slice dynamic CT scan [35], researches have managed to access capillary blood alteration using this method.

As we have shown in Table 1, reduced portal and total perfusion was observed in fibrosis models, relatively compensated by increase of hepatic arterial flow. The blockage of liver microcirculation due to collagen deposit and active remodeling may increase intrahepatic vascular resistance and exacerbate the disease progression. We demonstrated that portal venous ADMSC implantation benefited liver fibrosis by significantly increasing PVP and TLP (Table 2). FVIII staining and quantification confirmed the significant decrease of microvessel counts after ADMSC portal venous transplantation, supporting the idea that angiogenesis in CCl_4_ induced fibrosis model could be reversed by ADMSC treatment. The therapeutic mechanism of MSC for preventing liver fibrosis is not clear. Some have reported its function by effecting MMPs [34], or by suppressing inflammation and immunoreaction [36]. Importantly, MSCs were described in previous study having the ability to modulate VEGF delivery, differentiate into endothelial-like cells and anastomose with the host vasculature [37-40]. We observed significantly increased expression of VEGF at mRNA and protein level in fibrosis models, and the suppression of fibrosis by ADMSCs transplantation resulted in down regulation of VEGF. VEGF is one of the most central factors of angiogenesis and sinusoidal capillarisation in liver fibrosis [41,42]. VEGF and its receptor intensively expressed and interacted with activated HSC (hepatic stellate cell), which plays an important role in liver fibrosis development [43-45]. Extensive fibrosis related to hypoxemia or less blood supply may be a strong stimulator to VEGF production [46]. And decrease fibrosis contributing to modification of liver bloody supply may be accompanied by VEGF down regulation [47]. Our data correlated the angiogenesis marker VEGF to microcirculation improvement exhibited on CT perfusion scan, which helped to explain the underlying mechanism of fibrosis attenuation caused by ADMSC implantation.

There are still controversies about the most appropriate route of MSC transplantation. In our study, the effectiveness of different transplantation routes was compared. Kim SJ and co-workers have reported that tail vein transplantation was more effective than portal vein transplantation [48]. However, in our case, portal vein ADMSC implantation was documented significantly more blood supply alteration as that of tail vein implantation in CT perfusion scan. As is also revealed in histological findings, tail vein group had no improvement in fibrogenesis, but only reduced steatosis lesions, while portal vein group exhibited much more therapeutic potential. In the case of tail vein transplantation for MSC grafting, stem cells may not concentrate and disperse throughout the target organ, thus significantly less MSCs would reach liver and induce therapeutic effect in the target organ [49]. Portal venous infusion helped to mechanically trap grafted cells when they particularly present along sinusoids [48], thus raise the efficiency of ADMSC transplantation.

The efficacy and feasibility of MSC portal vein transfusion has been reported by other researches [50,51]. And as we observed, the results demonstrated a therapeutic advantage of intraportal infusion over peripheral venous infusion.

As discussed above, our data suggested that ADMSCs was easily accessible and expandable. Transplantation via portal vein gave their ability to improve microcirculation and ameliorate liver fibrosis. Future work will be required to explore deeply into the mechanism of how ADMSCs reduce fibrosis and evaluate the therapeutic potential of ADMSCs treatment in clinical settings.

## Conclusion

In conclusion, the present study suggested a protective role of ADMSCs in liver fibrosis, evidenced by increasing portal and total blood supply viewed in CT perfusion scan, decreased VEGF expression at mRNA and protein level, as well as improved liver function tests and hepatic biopsy. CT perfusion scan was a sensitive and non-invasive method to monitor therapeutic effect in recipient liver. In addition, we have also found that intraportal transplantation was a more appropriate pathway than tail vein transplantation.

## Competing interests

The authors declare that they have no competing interests.

## Authors’ contributions

WY and LF carried out the molecular genetic studies, animal experiments, participated in the sequence alignment and drafted the manuscript. WY and LF equally contributed to this publication. LJ participated in the animal experiments. FW carried out the immunoassays. XH helped to draft the manuscript. LL and YX performed the statistical analysis. CW ran the qPCR. YJ participated in the design of the study, coordinated and helped to draft the manuscript. All authors read and approved the final manuscript.
